# Cardiovascular Comorbidities and Advanced Chronic Kidney Disease in Hospitalized Patients with Multiple Myeloma: A Single-Center Retrospective Cohort Study

**DOI:** 10.3390/diseases14060214

**Published:** 2026-06-12

**Authors:** Lavinia Alice Bălăceanu, Andreea Taisia Tiron, Ion Daniel Baboi, Claudia Georgeta Iacobescu, Beatrice Bălăceanu-Gurău, Cristian-Dorin Gurău, Ioana Valeria Grigorescu, Ion Dina

**Affiliations:** 1 Clinical Department 1—Medical Semiology, “Carol Davila” University of Medicine and Pharmacy, 020021 Bucharest, Romania; alice.balaceanu@umfcd.ro (L.A.B.); andreea.tiron@umfcd.ro (A.T.T.); daniel.baboi@umfcd.ro (I.D.B.); ioana-valeria.grigorescu@rez.umfcd.ro (I.V.G.); ion.dina@umfcd.ro (I.D.); 2 Internal Medicine Clinic, “Sf. Ioan” Clinical Emergency Hospital, 042122 Bucharest, Romania; 3 Cardiology Clinic, “Sf. Ioan” Clinical Emergency Hospital, 042122 Bucharest, Romania; 4 Gastroenterology Clinic, “Sf. Ioan” Clinical Emergency Hospital, 042122 Bucharest, Romania; iacobescu_clodi@yahoo.com; 5 “Carol Davila” University of Medicine and Pharmacy, Orthopedics and Traumatology Clinic, Clinical Emergency Hospital, 020021 Bucharest, Romania

**Keywords:** multiple myeloma, advanced chronic kidney disease, cardiovascular comorbidities, atrial fibrillation, myocardial ischemia, hospitalized patients

## Abstract

Background: Advanced chronic kidney disease (CKD) and cardiovascular comorbidities frequently coexist in patients with multiple myeloma and are particularly common among hospitalized patients. However, the relationship between common cardiovascular comorbidities and advanced CKD in routine clinical practice remains incompletely characterized. Methods: We conducted a retrospective single-center cohort study including 137 hospitalized patients diagnosed with multiple myeloma between January 2015 and February 2026. Demographic, clinical, and laboratory data were extracted from electronic medical records. Advanced CKD was defined as eGFR < 30 mL/min/1.73 m^2^, calculated using the CKD-EPI 2021 equation. Patients with isolated acute kidney injury were excluded. Cross-sectional associations between cardiovascular comorbidities and advanced CKD were assessed using logistic regression models. Results: The median age was 69 years (IQR 63–77), and 56.9% of patients were women. Renal impairment was common, with a median creatinine level of 2.82 mg/dL and a median eGFR of 22.4 mL/min/1.73 m^2^. Advanced CKD was identified in 55 of 116 patients (47.4%) with available CKD classification. Cardiovascular comorbidities were common, including hypertension (42/55, 76.4%), diabetes mellitus (18/55, 32.7%), myocardial ischemia (41/55, 74.5%), and heart failure (25/55, 45.5%). In univariate analysis, atrial fibrillation showed a significant cross-sectional association with advanced CKD (OR 4.43, 95% CI 1.30–15.07, *p* = 0.017), as was myocardial ischemia (OR 2.89, 95% CI 1.07–7.80, *p* = 0.039). In multivariable analysis, atrial fibrillation demonstrated a trend toward an association with advanced CKD but did not remain statistically significant after adjustment. Conclusions: Advanced CKD and cardiovascular comorbidities frequently coexist in hospitalized patients with multiple myeloma. Atrial fibrillation and myocardial ischemia were associated with advanced CKD in univariate analyses; however, these associations were attenuated after multivariable adjustment. Overall, these findings provide insight into the coexistence of advanced CKD and cardiovascular comorbidities in hospitalized patients with multiple myeloma.

## 1. Introduction

Multiple myeloma is a hematological malignancy frequently associated with multi-organ involvement, particularly renal and cardiovascular complications, both of which significantly affect prognosis [1,2,3]. Kidney disease is a common and clinically relevant complication of multiple myeloma, ranging from mild CKD to dialysis-dependent renal failure [1,2,3]. Cardiovascular comorbidities are also highly prevalent, especially in elderly patients with advanced disease [4,5].

Although cardiac involvement, including amyloidosis, has been extensively studied, less is known about the relationship between common cardiovascular comorbidities—such as atrial fibrillation and myocardial ischemia—and advanced CKD in routine clinical practice [6,7,8,9,10,11,12]. The coexistence of cardiovascular comorbidities and advanced CKD may reflect shared clinical factors, including advanced age, systemic inflammation, and the presence of multiple comorbid conditions [13,14,15].

Cardiac involvement in multiple myeloma may include heart failure, arrhythmias, myocardial ischemia, and, in selected cases, cardiac amyloidosis [16,17,18]. Heart failure with preserved ejection fraction and atrial fibrillation are frequently encountered in light-chain cardiac amyloidosis [16,18]. In some cases, heart failure may occur secondary to an acute coronary event, despite the absence of obstructive coronary artery disease on angiography [19]. Atrial thrombosis and thromboembolism may occur due to atrial fibrillation or atrial electromechanical dissociation, even in patients with sinus rhythm [17]. Laboratory evaluation in multiple myeloma includes hematological, renal, and cardiac parameters, such as serum and urine protein electrophoresis, serum free light chains, serum creatinine, proteinuria, NT-proBNP, and troponins [20,21,22]. Cardiac biomarkers and transthoracic echocardiography are particularly useful in clinical practice; however, their interpretation may be influenced by comorbidities such as advanced CKD [18].

Given the frequent coexistence of renal and cardiovascular comorbidities in multiple myeloma, a more comprehensive characterization of these associations may enhance our understanding of the overall comorbidity burden among hospitalized patients.

The aim of this study was to evaluate the association between cardiovascular comorbidities and advanced CKD in hospitalized patients with multiple myeloma.

## 2. Materials and Methods

### 2.1. Study Design

This retrospective study was conducted on a cohort of 137 patients diagnosed with multiple myeloma and admitted to “Sf. Ioan” Clinical Emergency Hospital, Bucharest, Romania, between 1 January 2015 and 28 February 2026. Patient diagnoses were identified using both the Diagnosis-Related Group (DRG) system and free-text medical records, including primary and secondary diagnoses. Inclusion criteria comprised adults (≥18 years of age) diagnosed with multiple myeloma who were admitted during the study period, with available clinical and laboratory data. Exclusion criteria included incomplete medical records, missing key variables, and duplicate admissions. Comorbidities were identified using a combination of ICD-10 coded diagnoses, discharge summaries, medication records, and clinician-documented diagnoses available in the electronic medical records. To avoid duplicate counting, repeated records referring to the same chronic condition were consolidated during data processing. Nevertheless, because comorbidity ascertainment relied on retrospective review of administrative and clinical records, some degree of coding bias and overestimation cannot be excluded.

Advanced CKD was defined as eGFR < 30 mL/min/1.73 m^2^. Estimated glomerular filtration rate (eGFR) was routinely available in the hospital electronic medical record system and was automatically reported by the institutional laboratory using the CKD-EPI 2021 equation. CKD classification was based on both documented pre-existing CKD diagnoses recorded in the medical history and renal function parameters obtained during hospitalization. CKD classification was available for 116 patients. Quantitative eGFR values were available for 112 patients, while in four additional patients CKD status could be established based on documented medical history and clinical records despite the absence of a recorded eGFR value in the study database. Patients with isolated acute kidney injury in the absence of underlying CKD were excluded. In cases without a previously documented diagnosis of CKD, renal function was reassessed during hospitalization using serial serum creatinine measurements and evaluation at discharge after treatment of potentially reversible causes of renal dysfunction. Previous medical records were also reviewed whenever available to confirm the chronic nature of renal impairment and reduce the risk of misclassifying transient renal dysfunction as chronic kidney disease. This definition was selected to identify clinically significant renal impairment within a cohort characterized by a high prevalence of CKD. Secondary analyses explored associations between cardiovascular comorbidities and the presence of CKD (all stages).

Myocardial ischemia was defined based on documented medical history recorded in patient files, including prior diagnosis of ischemic heart disease, previous myocardial infarction, or angina, as noted in discharge summaries or cardiology evaluations.

### 2.2. Data Collection

Demographic data (age, gender) and clinical variables were collected. Age was analyzed both as a continuous and categorical variable, divided into four groups: <60 years, 60–70 years, 70–80 years, and ≥80 years. The dataset included 70 variables per patient, encompassing hematological, biochemical, immunological, and clinical parameters.

### 2.3. Statistical Analysis

Normality was assessed using the Shapiro–Wilk test (*p* < 0.05 indicating non-normal distribution). Continuous variables are presented as median and interquartile range (IQR), while categorical variables are presented as frequencies and percentages. Group comparisons were performed using the Mann–Whitney U test for continuous variables and the chi-square test or Fisher’s exact test for categorical variables, as appropriate. Univariate logistic regression analyses were performed to evaluate cross-sectional associations between individual cardiovascular comorbidities and advanced CKD. Odds ratios (ORs) with 95% confidence intervals were calculated for each variable. A *p*-value < 0.05 was considered statistically significant. To evaluate factors associated with advanced CKD, a multivariable logistic regression model was constructed using maximum likelihood estimation. Independent variables included age, sex, atrial fibrillation, and myocardial ischemia. Only variables with sufficient data completeness were considered for inclusion in the adjusted model. Diabetes mellitus and hypertension were not included in the final adjusted model due to a high proportion of missing data and reduced model stability. The multivariable analysis was performed using complete-case data. Missing values were not imputed and were not considered equivalent to the absence of a diagnosis. Variables with substantial missing data were excluded from the adjusted model to preserve sample size and model stability. The number of patients included in the adjusted model and the number of advanced CKD events are reported in the Results Section. Given the limited sample size and number of events, the adjusted model was considered exploratory.

## 3. Results

### 3.1. Demographic and Clinical Characteristics

**Figure 1:**
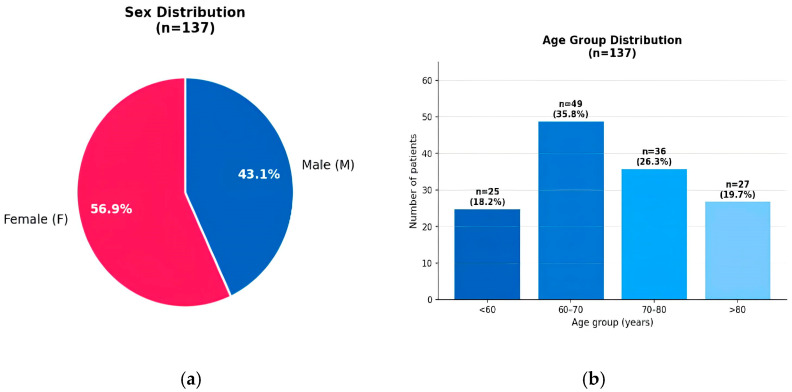
(**a**) Distribution by gender and (**b**) age group.

The study included 137 patients, of whom 78 were women (56.9%) and 59 were men (43.1%), with a female-to-male ratio of 1.32:1. The median age was 69 years (IQR 63–77), with the majority of patients (62%) aged between 60 and 80 years. The youngest patient was 42 years old, whereas the oldest was 91 years old. Patients under 60 years accounted for 18.2% of the cohort, while 19.7% were over 80 years old, confirming that multiple myeloma predominantly affects older individuals (Figure 1).

The myeloma subtype was identified in 44.5% of patients, with IgG being the most frequent subtype (63.9%).

### 3.2. Biological Profile

The main biological parameters are presented in Table 1. The median hemoglobin level was 9.3 g/dL, indicating frequent anemia. Median creatinine was 2.82 mg/dL, and median eGFR (CKD-EPI 2021) was 22.4 mL/min/1.73 m^2^, reflecting substantial renal impairment in a large proportion of patients.

All analyzed variables showed non-normal distributions, supporting the use of non-parametric statistical methods (Table 1).

### 3.3. Comorbidity Profile

**Figure 2:**
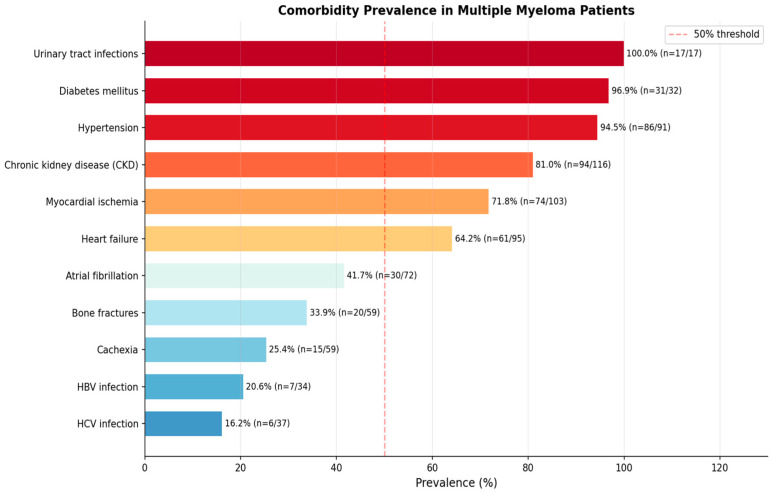
Prevalence of comorbidities. Percentages were calculated using the number of patients with available information for each specific comorbidity. Because data completeness varied across variables, denominators differ and prevalence estimates should be interpreted accordingly.

The comorbidity profile demonstrated the frequent coexistence of metabolic and cardiovascular conditions within the study cohort. Comorbidity information was extracted for the entire cohort; however, data completeness varied across individual variables. Among the 116 patients with available CKD classification, CKD was identified in 94 (81.0%). Quantitative eGFR values were available for 112 of these patients. Among the 94 patients with CKD, 55 (58.5%) had advanced CKD, defined as an eGFR < 30 mL/min/1.73 m^2^. Therefore, advanced CKD was identified in 55 of the 116 evaluable patients (47.4%). For the purpose of logistic regression analyses, advanced CKD status was evaluated in the entire subset of 116 patients with available CKD classification, yielding 55 cases (47.4%) and 61 non-cases. These findings likely reflect the advanced age of the study population, the substantial comorbidity burden, and the hospital-based nature of the cohort (Figure 2).

Among patients with advanced CKD, diabetes mellitus was identified in 18/55 (32.7%) and hypertension in 42/55 (76.4%). The prevalence estimates for individual comorbidities should be interpreted in the context of variable data completeness across medical records. The number of patients with available information differed substantially across comorbidities, ranging from 32 patients for diabetes mellitus to 116 patients for CKD. Consequently, prevalence estimates based on available-case denominators may overestimate the frequency of certain conditions when compared with calculations using the entire cohort as the denominator.

Comorbidities were categorized into three groups:

Very high prevalence (>80%): diabetes, hypertension, CKD
High prevalence (50–79%): myocardial ischemia, heart failure
Moderate prevalence (<50%): atrial fibrillation, fractures, cachexia

### 3.4. Comparative Analysis by Sex

eGFR CKD-EPI 2021 (mL/min/1.73 m^2^) was significantly lower in women than in men (*p* = 0.012), while INR values were significantly higher in men (*p* = 0.024). No other statistically significant sex-related differences were observed among the analyzed biological parameters.

### 3.5. Cardiovascular Associations with Advanced CKD

Among the 116 patients with available CKD classification, CKD was identified in 94 patients (81.0%). Advanced CKD, defined as an eGFR <30 mL/min/1.73 m^2^, was identified in 55/116 patients (47.4%). In univariate analyses, atrial fibrillation was significantly associated with advanced CKD (OR 4.43, 95% CI 1.30–15.07, *p* = 0.017). Myocardial ischemia was also significantly associated with advanced CKD (OR 2.89, 95% CI 1.07–7.80, *p* = 0.039). However, both associations were attenuated after multivariable adjustment, and statistical significance was not maintained. Heart failure was associated with a non-significant increase in the odds of advanced CKD.

Univariate logistic regression analyses were performed to evaluate cross-sectional associations between cardiovascular comorbidities and advanced CKD. These findings suggest that the associations observed in univariate analyses may be partly explained by clinical factors that were not fully captured in the adjusted model (Table 2).

A multivariable logistic regression model was performed as an exploratory analysis to assess cross-sectional associations between selected cardiovascular comorbidities and advanced CKD (Table 3). The analysis was based on complete-case data and included 116 patients with available CKD classification and complete information for all variables retained in the final model, of whom 55 met the definition of advanced CKD. The adjusted model included age, sex, atrial fibrillation, and myocardial ischemia. Diabetes mellitus and hypertension were excluded because of substantial missing data, which would have reduced model stability. Missing values were not imputed and were not recoded as the absence of disease. Given the limited sample size and number of events, the results of the adjusted model should be interpreted as exploratory.

Specific cardiovascular conditions, particularly atrial fibrillation and myocardial ischemia, showed cross-sectional associations with advanced CKD in patients with multiple myeloma. In the adjusted model, atrial fibrillation demonstrated a trend toward association with advanced CKD (aOR 3.14, 95% CI 0.89–11.13, *p* = 0.076), although statistical significance was not maintained after adjustment. Myocardial ischemia was not independently associated with advanced CKD after multivariable adjustment (aOR 0.64, 95% CI 0.18–2.24, *p* = 0.482). Age was not significantly associated with advanced CKD, while male sex showed a borderline inverse association (aOR 0.36, 95% CI 0.13–1.01, *p* = 0.053).

## 4. Discussion

In this retrospective cohort of hospitalized patients with multiple myeloma, advanced chronic kidney disease and cardiovascular comorbidities were highly prevalent. Atrial fibrillation and myocardial ischemia were associated with advanced CKD in univariate analysis, although these associations were attenuated after multivariable adjustment. These findings suggest that cardiovascular comorbidities commonly coexist with advanced CKD in this patient population. The observed associations should not be interpreted as evidence that cardiovascular comorbidities contribute directly to the development or progression of advanced CKD, nor that advanced CKD predisposes to these cardiovascular conditions. Rather, they highlight patterns of coexistence between cardiovascular and renal comorbidities within a hospitalized multiple myeloma population.

Our findings are consistent with previous reports describing the coexistence of cardiovascular and renal involvement in multiple myeloma [23,24,25]. The high prevalence of advanced CKD is consistent with the established pathophysiology of multiple myeloma, where light chain deposition contributes to kidney injury [25]. Similarly, the high prevalence of cardiovascular comorbidities reflects the systemic nature of the disease and its complications [4,5]. The high prevalence of cardiovascular comorbidities observed in our cohort is consistent with the demographic and clinical profile typically seen in patients with multiple myeloma, a disease that predominantly affects older adults. Traditional cardiovascular risk factors, such as hypertension and dyslipidemia, together with myeloma-related conditions including anemia, hyperviscosity, thrombosis, renal dysfunction, and treatment-related adverse effects, may contribute to cardiovascular complications in this population [4]. Hypertension, in particular, may influence myocardial structure and vascular function independently of the underlying hematologic malignancy [4]. Accordingly, the coexistence of multiple cardiovascular and renal comorbidities in hospitalized patients with multiple myeloma is likely driven by a complex interplay of age-related, hemodynamic, metabolic, and disease-related factors [4]. The coexistence of atrial fibrillation and advanced CKD may reflect shared clinical and pathophysiological factors. However, the association was attenuated after multivariable adjustment and should therefore be interpreted cautiously. Myocardial ischemia may coexist with advanced CKD in patients with a high burden of cardiovascular comorbidities [19,22,26,27].

The multivariable analysis attenuated the associations observed in univariate analysis, suggesting that cardiovascular comorbidities may represent markers of overall clinical complexity rather than independent determinants of advanced CKD. Although atrial fibrillation demonstrated a trend toward association with advanced CKD after adjustment, statistical significance was not maintained. The findings of the multivariable analysis should be interpreted with caution. Because of the relatively small sample size, the limited number of events, and the high prevalence of diabetes mellitus and hypertension, adjustment for all potentially relevant confounders was not feasible. Consequently, residual confounding cannot be excluded. The adjusted model should be regarded as exploratory and not as providing definitive evidence of independent associations between cardiovascular comorbidities and advanced CKD. The associations observed in this study may reflect shared clinical characteristics, including advanced age, systemic disease severity, and the frequent coexistence of multiple cardiovascular and renal comorbidities. However, due to the retrospective design and limited sample size, causal relationships cannot be established. These findings may help characterize the coexistence of cardiovascular and renal comorbidities in hospitalized patients with multiple myeloma. Renal dysfunction in older patients with multiple myeloma is frequently the result of complex and multifactorial pathophysiological processes [25]. The respective contributions of multiple myeloma and common comorbidities, including chronic kidney disease and diabetes mellitus, may be difficult to disentangle, particularly in hospitalized patients with multiple coexisting conditions [25]. Consequently, the diagnosis and characterization of renal impairment may be challenging and occasionally delayed [25].

The very high prevalence of hypertension and diabetes mellitus observed in our cohort should be interpreted within the context of the study setting. Our institution is a tertiary emergency referral center that predominantly manages elderly patients with advanced multiple myeloma and multiple chronic comorbidities. Furthermore, the study population consisted exclusively of hospitalized patients, which differs substantially from population-based or outpatient cohorts. The cohort was therefore enriched for individuals with a particularly high burden of cardiovascular and metabolic comorbidities. Comorbidities were identified through review of both administrative records and electronic medical records. Although diagnoses were carefully reviewed and duplicate entries were excluded during data validation, retrospective ascertainment may still be influenced by coding practices and documentation patterns. Therefore, the reported prevalence estimates should be interpreted as reflecting the characteristics of a highly selected hospitalized population rather than the broader multiple myeloma population. In particular, the apparently very high prevalence of diabetes mellitus (96.9%), hypertension (94.5%), atrial fibrillation (41.7%), myocardial ischemia (71.8%), and heart failure (64.2%) reflects the use of available-case denominators and should not be interpreted as prevalence estimates for the entire cohort. When calculated using the entire cohort as the denominator (n = 137), the prevalence estimates were lower than those calculated using available-case denominators, highlighting the impact of missing data on descriptive prevalence estimates. For example, diabetes mellitus was identified in 31 patients (22.6% of the entire cohort), hypertension in 86 patients (62.8%), myocardial ischemia in 74 patients (54.0%), and heart failure in 61 patients (44.5%). Previous studies have suggested that early identification of patients with advanced CKD and cardiovascular disease may facilitate closer clinical monitoring and individualized therapeutic strategies [28,29,30]. Identification of chronic kidney disease preceding the diagnosis of the hematologic malignancy may assist in distinguishing smoldering multiple myeloma from symptomatic multiple myeloma [28]. The present study was not designed to evaluate these clinical outcomes.

Cardiac amyloidosis is an important cardiovascular complication of multiple myeloma and may contribute to both cardiac and renal dysfunction [18,20,31,32,33,34,35,36]. Current guidelines support the diagnosis and management of cardiac amyloidosis, considering advanced age and multiple comorbidities [32,33,34,35,36]. In our cohort, systematic cardiac evaluation using echocardiography, cardiac magnetic resonance imaging, and standardized cardiac biomarkers was not consistently available. Therefore, the contribution of cardiac amyloidosis to the observed cardiovascular and renal manifestations could not be directly assessed and remains speculative. Future prospective studies incorporating standardized cardiac evaluation are needed to better characterize cardiac involvement in hospitalized patients with multiple myeloma.

Several limitations should be acknowledged. The retrospective single-center design may introduce selection bias and limit external validity. The study population was derived from a single tertiary care center and consisted exclusively of hospitalized patients. Cardiovascular comorbidities were identified based on medical records rather than standardized diagnostic protocols, which may introduce misclassification bias. Although multivariable analysis was performed, the relatively small sample size and missing data limited the stability and complexity of the adjusted model. Because cardiovascular comorbidities and renal status were assessed during the same hospitalization period, the temporal sequence between these conditions could not be established. Therefore, the observed relationships should be interpreted as descriptive cross-sectional associations rather than evidence of causality or disease progression. The multivariable model should be interpreted as exploratory because the statistical power was limited by the sample size. To reduce the risk of model overfitting, the number of predictors was restricted according to the commonly recommended rule of approximately 10 events per predictor variable.

Although patients with isolated acute kidney injury were excluded and CKD classification incorporated documented medical history and serial renal assessments during hospitalization, some degree of misclassification cannot be completely excluded because of the retrospective nature of the study.

Despite these limitations, the study provides real-world data on the coexistence of cardiovascular and renal comorbidities in hospitalized patients with multiple myeloma and may inform future prospective investigations in this field. These findings should be interpreted as cross-sectional observations derived from a tertiary-care cohort and may provide a foundation for future prospective investigations.

Future prospective studies using standardized definitions and data collection protocols are needed to better characterize the coexistence of renal and cardiovascular comorbidities in patients with multiple myeloma. The present findings should primarily be interpreted as describing the coexistence of advanced CKD and cardiovascular comorbidities within a hospitalized multiple myeloma cohort. Broader considerations regarding cardiac amyloidosis and specialized cardio-oncology evaluation extend beyond the scope of the current dataset and warrant dedicated prospective investigation. Standardized cardiac evaluation, systematic assessment for amyloidosis, and larger prospective cohorts are needed to confirm these findings and further characterize cardiovascular and renal comorbidities in patients with multiple myeloma.

## 5. Conclusions

In this single-center retrospective cohort of hospitalized patients with multiple myeloma, advanced CKD and cardiovascular comorbidities were highly prevalent. Atrial fibrillation and myocardial ischemia were associated with advanced CKD in univariate analyses, but these associations were attenuated after multivariable adjustment, suggesting that they may reflect broader clinical complexity rather than independent determinants of advanced CKD. Given the relatively limited sample size and the attenuation of the observed associations following multivariable adjustment, larger prospective studies incorporating standardized renal and cardiovascular assessments are warranted to validate these findings and further elucidate the associations between cardiovascular comorbidities and advanced CKD in patients with multiple myeloma.

## Figures and Tables

**Table 1:** Main descriptive statistics.

Parameter	*N*	Mean ± SD	Median [IQR]	Min–Max
Age (years)	137	69.4 ± 10.8	69 [63–77]	42–91
Hemoglobin (g/dL)	116	9.33 ± 2.59	9.3 [7.5–11.2]	4.1–17.2
Neutrophile (×10^3^/μL)	104	6645 ± 6527	5235 [3243–7361]	842–47,550
NLR	104	7.05 ± 8.16	3.88 [2.62–7.43]	0.73–53.7
Creatinine (mg/dL)	112	4.26 ± 4.00	2.82 [1.30–6.34]	0.52–20.7
eGFR (CKD-EPI 2021) (mL/min/1.73 m^2^)	112	36.0 ± 32.8	22.4 [8.6–46.1]	2.1–142.8
CRP (mg/L)	75	46.1 ± 79.6	13.1 [3.0–43.6]	0.12–432
Serum albumin (g/dL)	61	3.42 ± 0.86	3.32 [2.82–4.14]	1.60–5.20
INR	88	1.39 ± 0.61	1.23 [1.08–1.46]	0.92–4.93
IgG (mg/dL)	39	2450 ± 2469	1334 [485–4635]	28–8341

**Table 2:** Univariate logistic regression analysis of cardiovascular comorbidities and advanced CKD.

Associated Comorbidities	OR	95% CI	*p*-Value
Atrial fibrillation	4.43	1.30–15.07	0.017
Myocardial ischemia	2.89	1.07–7.80	0.039
Heart failure	2.73	1.00–7.44	0.071

OR = odds ratio; CI = confidence interval. Odds ratios were derived from univariate logistic regression analyses.

**Table 3:** Multivariable logistic regression analysis for advanced CKD.

Variable	Adjusted OR (aOR)	95% CI	*p*-Value
Age	1.01	0.96–1.07	0.720
Male sex	0.36	0.13–1.01	0.053
Atrial fibrillation	3.14	0.89–11.13	0.076
Myocardial ischemia	0.64	0.18–2.24	0.482

## Data Availability

The original contributions presented in this study are included in the article. Further inquiries can be directed to the corresponding authors.

## Abbreviations

The following abbreviations are used in this manuscript:

CKD	Chronic Kidney Disease
CRP	C-reactive protein
ECG	Electrocardiogram
HBV	Hepatitis B virus
HCV	Hepatitis C virus
IgG	Immunoglobulin G
INR	International normalized ratio
NLR	Neutrophil-to-lymphocyte ratio
NT-proBNP	N-terminal pro-B-type natriuretic peptide
NYHA	New York Heart Association

## References

[1] Rajkumar S.V., Kumar S. (2025). Monoclonal Gammopathy of Undetermined Significance. N. Engl. J. Med..

[2] Ikura H., Endo J., Kitakata H., Moriyama H., Sano M., Fukuda K. (2022). Molecular Mechanism of Pathogenesis and Treatment Strategies for AL Amyloidosis. Int. J. Mol. Sci..

[3] Sutanto H., Romadhon P.Z., Fatmawati V.R., Waitupu A., Ansharullah B.A., Rachma B., Elisa E., Pratiwi L., Adytia G.J. (2025). Multiple Myeloma and Precursor Plasma Cell Disorders: From Emerging Driver Mutations to Current and Future Therapeutic Strategies. Hemato.

[4] Colomba A., Astarita A., Mingrone G., Airale L., Catarinella C., Vallelonga F., Leone D., Cesareo M., Paladino A., Bringhen S. (2024). Haemodynamic Forces: Emerging Markers of Ventricular Remodelling in Multiple Myeloma Cardiovascular Baseline Risk Assessment. Cancers.

[5] Fontes Oliveira M., Naaktgeboren W.R., Hua A., Ghosh A.K., Oakervee H., Hallam S., Manisty C. (2021). Optimising cardiovascular care of patients with multiple myeloma. Heart.

[6] Tothong W., Tantiworawit A., Norasetthada L., Chai-Adisaksopha C., Punnachet T., Hantrakun N., Piriyakhuntorn P., Rattanathammethee T., Hantrakool S., Rattarittamrong E. (2024). Prevalence, Outcomes and Impact of Disease-Related Complications in the Survival of Multiple Myeloma Patients. Hematol. Rep..

[7] Moltó-Balado P., Clua-Espuny J.L., Tarongi-Vidal C., Barrios-Carmona P., Alonso-Barberán V., Balado-Albiol M.T., Simeó-Monzó A., Canela-Royo J., Del Barrio-González A. (2025). Risk of Chronic Kidney Disease and Implications in Patients with Atrial Fibrillation for the Development of Major Adverse Cardiovascular Events with Machine Learning. Med. Sci..

[8] Suliman I.L., Panculescu F.G., Cimpineanu B., Popescu S., Fasie D., Cozaru G.C., Gafar N., Tuta L.A., Alexandru A. (2025). The Interplay of Cardiovascular Comorbidities and Anticoagulation Therapy in ESRD Patients on Haemodialysis-The South-Eastern Romanian Experience. Biomedicines.

[9] Stefani G., Kouvata E., Vassilopoulos G. (2023). Light-Chain Amyloidosis: The Great Impostor. Life.

[10] Sabinot A., Ghetti G., Pradelli L., Bellucci S., Lausi A., Palladini G. (2023). State-of-the-art review on AL amyloidosis in Western Countries: Epidemiology, health economics, risk assessment and therapeutic management of a rare disease. Blood Rev..

[11] Triposkiadis F., Briasoulis A., Xanthopoulos A. (2024). Amyloids and the Heart: An Update. J. Clin. Med..

[12] Ríos-Tamayo R., Krsnik I., Gómez-Bueno M., Garcia-Pavia P., Segovia-Cubero J., Huerta A., Salas C., Silvestre R.A., Sánchez A., Manso M. (2023). AL Amyloidosis and Multiple Myeloma: A Complex Scenario in Which Cardiac Involvement Remains the Key Prognostic Factor. Life.

[13] Aureli A., Marziani B., Sconocchia T., Pasqualone G., Franceschini L., Spagnoli G.C., Venditti A., Sconocchia G. (2025). Challenges in Multiple Myeloma Therapy in Older and Frail Patients. Cancers.

[14] Mukkamalla S.K.R., Malipeddi D. (2021). Myeloma Bone Disease: A Comprehensive Review. Int. J. Mol. Sci..

[15] Wechalekar A.D., Fontana M., Quarta C.C., Liedtke M. (2022). AL Amyloidosis for Cardiologists: Awareness, Diagnosis, and Future Prospects: *JACC: CardioOncology* State-of-the-Art Review. JACC CardioOncol..

[16] Aimo A., Ferrari Chen Y.F., Chianca M., Mori F., Pucci A., Castiglione V., Musetti V., Caponi L., Fabiani I., Cardinale D. (2025). Serum and Tissue Light-Chains as Disease Biomarkers in AL Amyloidosis. Int. J. Mol. Sci..

[17] Holcman K., Ząbek A., Boczar K., Podolec P., Kostkiewicz M. (2024). Management of Arrhythmias and Conduction Disorders in Amyloid Cardiomyopathy. J. Clin. Med..

[18] Palladini G., Schönland S., Merlini G., Milani P., Jaccard A., Bridoux F., Dimopoulos M.A., Ravichandran S., Hegenbart U., Roeloffzen W. (2023). The management of light chain (AL) amyloidosis in Europe: Clinical characteristics, treatment patterns, and efficacy outcomes between 2004 and 2018. Blood Cancer J..

[19] Tsai S.B., Seldin D.C., Wu H., O’Hara C., Ruberg F.L., Sanchorawala V. (2011). Myocardial infarction with "clean coronaries" caused by amyloid light-chain AL amyloidosis: A case report and literature review. Amyloid.

[20] De Michieli L., Cipriani A., Iliceto S., Dispenzieri A., Jaffe A.S. (2024). Cardiac Troponin in Patients with Light Chain and Transthyretin Cardiac Amyloidosis: *JACC: CardioOncology* State-of-the-Art Review. JACC CardioOncol..

[21] Gremese E., Bruno D., Varriano V., Perniola S., Petricca L., Ferraccioli G. (2023). Serum Albumin Levels: A Biomarker to Be Repurposed in Different Disease Settings in Clinical Practice. J. Clin. Med..

[22] Padala S.A., Barsouk A., Barsouk A., Rawla P., Vakiti A., Kolhe R., Kota V., Ajebo G.H. (2021). Epidemiology, Staging, and Management of Multiple Myeloma. Med. Sci..

[23] Charliński G., Steinhardt M., Rasche L., Gonzalez-Calle V., Peña C., Parmar H., Wiśniewska-Piąty K., Vallas J.D., Olszewska-Szopa M., Jurczyszyn A. (2024). Outcomes of Modified Mayo Stage IIIa and IIIb Cardiac Light-Chain Amyloidosis: Real-World Experience in Clinical Characteristics and Treatment-67 Patients Multicenter Analysis. Cancers.

[24] Allegra A., Mirabile G., Tonacci A., Genovese S., Pioggia G., Gangemi S. (2023). Machine Learning Approaches in Diagnosis, Prognosis and Treatment Selection of Cardiac Amyloidosis. Int. J. Mol. Sci..

[25] Zorlu T., Kayer M.A., Okumus N., Ulaş T., Dal M.S., Altuntas F. (2025). Challenges, Difficulties, and Delayed Diagnosis of Multiple Myeloma. Diagnostics.

[26] Gavriatopoulou M., Paschou S.A., Ntanasis-Stathopoulos I., Dimopoulos M.A. (2021). Metabolic Disorders in Multiple Myeloma. Int. J. Mol. Sci..

[27] Morè S., Corvatta L., Manieri V.M., Morsia E., Offidani M. (2024). The Challenging Approach to Multiple Myeloma: From Disease Diagnosis and Monitoring to Complications Management. Cancers.

[28] Dimopoulos M.A., Terpos E., Boccadoro M., Moreau P., Mateos M.V., Zweegman S., Cook G., Engelhardt M., Delforge M., Hajek R. (2025). EHA-EMN Evidence-Based Guidelines for diagnosis, treatment and follow-up of patients with multiple myeloma. Nat. Rev. Clin. Oncol..

[29] Bergstrom D.J., Kotb R., Louzada M.L., Sutherland H.J., Tavoularis S., Venner C.P., Côté J., LeBlanc R., Reiman A., Sebag M. (2020). Consensus Guidelines on the Diagnosis of Multiple Myeloma and Related Disorders: Recommendations of the Myeloma Canada Research Network Consensus Guideline Consortium. Clin. Lymphoma Myeloma Leuk..

[30] Pinney J., Roufosse C., Kousios A., Chaidos A., Gillmore J.D., Rainone F., Choudhuri S., Ramasamy K., Blakey S., Ashcroft J. (2025). Diagnosis and management of monoclonal gammopathy of renal significance: A British Society for Haematology good practice paper. Br. J. Haematol..

[31] Hummel K., Meawad H., Gunning W.T., Gohara A.F. (2021). Negative Fat Pad Biopsy in Systemic AL: A Case Report Analyzing the Preferred Amyloidosis Screening Test. Diseases.

[32] Tan M., Chen Y., Ooi M., de Mel S., Tan D., Soekojo C., Nagarajan C. (2023). AL amyloidosis: Singapore Myeloma Study Group consensus guidelines on diagnosis, treatment and management. Ann. Acad. Med. Singap..

[33] Kitaoka H., Izumi C., Izumiya Y., Inomata T., Ueda M., Kubo T., Koyama J., Sano M., Sekijima Y., Tahara N. (2020). JCS 2020 Guideline on Diagnosis and Treatment of Cardiac Amyloidosis. Circ. J..

[34] Fine N.M., Davis M.K., Anderson K., Delgado D.H., Giraldeau G., Kitchlu A., Massie R., Narayan J., Swiggum E., Venner C.P. (2020). Canadian Cardiovascular Society/Canadian Heart Failure Society Joint Position Statement on the Evaluation and Management of Patients with Cardiac Amyloidosis. Can. J. Cardiol..

[35] Garcia-Pavia P., Rapezzi C., Adler Y., Arad M., Basso C., Brucato A., Burazor I., Caforio A.L.P., Damy T., Eriksson U. (2021). Diagnosis and treatment of cardiac amyloidosis: A position statement of the ESC Working Group on Myocardial and Pericardial Diseases. Eur. Heart J..

[36] Dorbala S., Ando Y., Bokhari S., Dispenzieri A., Falk R.H., Ferrari V.A., Fontana M., Gheysens O., Gillmore J.D., Glaudemans A.W.J.M. (2021). ASNC/AHA/ASE/EANM/HFSA/ISA/SCMR/SNMMI Expert Consensus Recommendations for Multimodality Imaging in Cardiac Amyloidosis: Part 1 of 2-Evidence Base and Standardized Methods of Imaging. Circ. Cardiovasc. Imaging.

